# Interactions between tumor-derived proteins and Toll-like receptors

**DOI:** 10.1038/s12276-020-00540-4

**Published:** 2020-12-09

**Authors:** Gun-Young Jang, Ji won Lee, Young Seob Kim, Sung Eun Lee, Hee Dong Han, Kee-Jong Hong, Tae Heung Kang, Yeong-Min Park

**Affiliations:** 1 Department of Immunology, College of Medicine, Konkuk University, 268 Chungwon-daero Chungju-si Chungcheongbuk-do, 27478 Seoul, South Korea; 2 R&DB Foundation, Konkuk University, 120 Neungdong-ro, Gwangjin-gu, Seoul, South Korea

**Keywords:** Immune cell death, Cancer microenvironment

## Abstract

Damage-associated molecular patterns (DAMPs) are danger signals (or alarmins) alerting immune cells through pattern recognition receptors (PRRs) to begin defense activity. Moreover, DAMPs are host biomolecules that can initiate a noninflammatory response to infection, and pathogen-associated molecular pattern (PAMPs) perpetuate the inflammatory response to infection. Many DAMPs are proteins that have defined intracellular functions and are released from dying cells after tissue injury or chemo-/radiotherapy. In the tumor microenvironment, DAMPs can be ligands for Toll-like receptors (TLRs) expressed on immune cells and induce cytokine production and T-cell activation. Moreover, DAMPs released from tumor cells can directly activate tumor-expressed TLRs that induce chemoresistance, migration, invasion, and metastasis. Furthermore, DAMP-induced chronic inflammation in the tumor microenvironment causes an increase in immunosuppressive populations, such as M2 macrophages, myeloid-derived suppressor cells (MDSCs), and regulatory T cells (Tregs). Therefore, regulation of DAMP proteins can reduce excessive inflammation to create an immunogenic tumor microenvironment. Here, we review tumor-derived DAMP proteins as ligands of TLRs and discuss their association with immune cells, tumors, and the composition of the tumor microenvironment.

## Cancer: Boosting the right signals to suppress tumors

Tumor cells killed by radiotherapy or chemotherapy release signaling molecules that stimulate both immune response and tumor aggressiveness; regulating these molecules could improve treatment efficacy. Tae Heung Kang, Yeong-Min Park, and co-workers at Konkuk University, Seoul, South Korea, have reviewed the role of damage-associated molecular patterns (DAMPs) in immunity and cancer. These signaling molecules act as danger signals, activating immune cells by binding to specific receptors. However, tumor cells have the same receptors, and DAMPs binding triggers chemoresistance and increases invasiveness. The researchers report that although DAMPs can trigger a helpful immune response, they can also cause chronic inflammation, which in turn promotes an immune suppression response, allowing tumors to escape immune detection. Improving our understanding of the functions of different DAMPs could improve our ability to boost the immune response and decrease tumor aggressiveness.

## Introduction

**Fig. 1:**
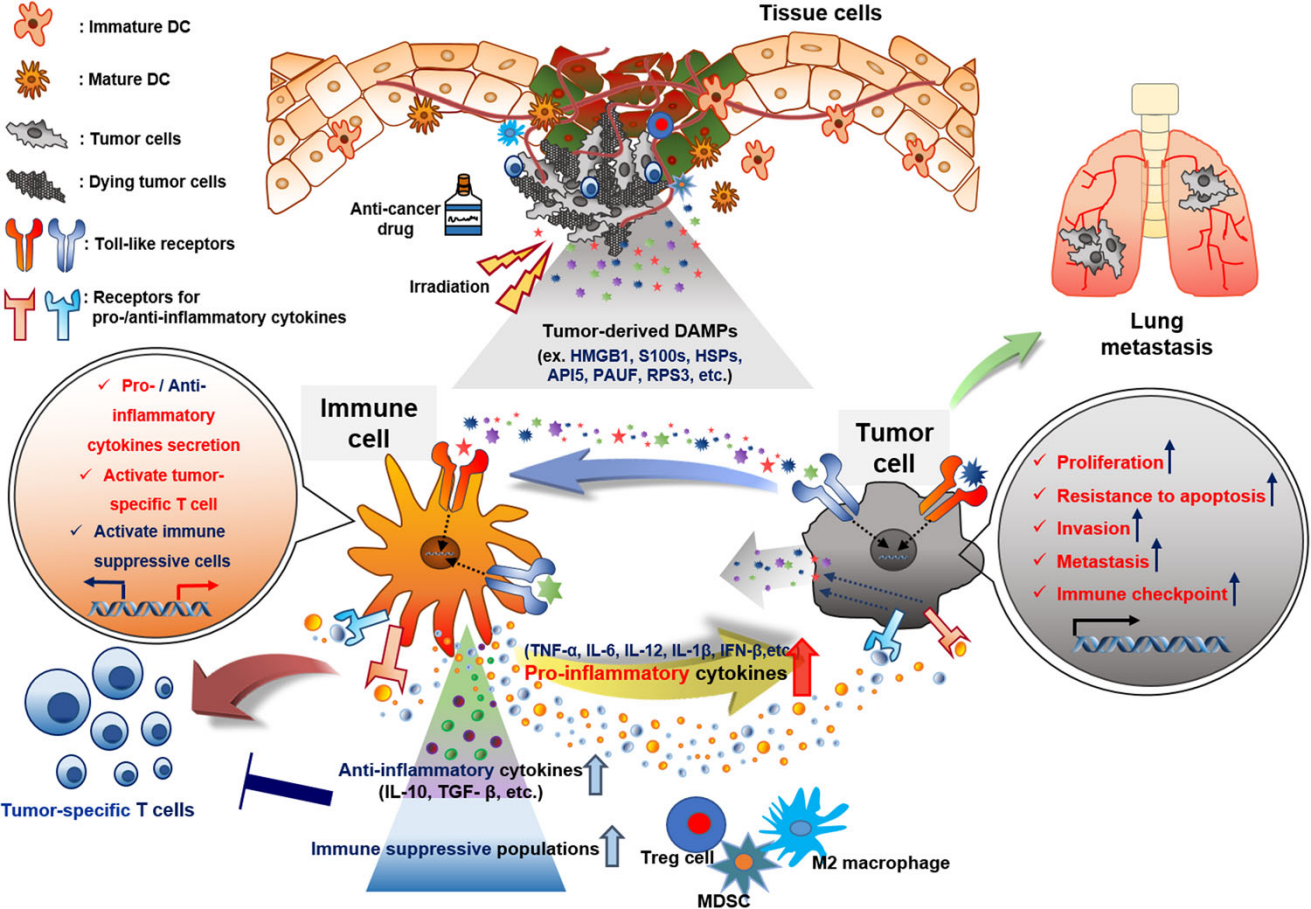
Interplay among DAMP proteins from dead cells, TLRs on immune cells, and cancer cells in the tumor microenvironment. Anticancer drugs and irradiation induce apoptosis or necrosis in tumor cells. Several DAMP proteins (HMGB1, S100, HSP, API5, PAUF, RPS3, etc.) released from dying tumor cells stimulate immune cells or tumor cells after binding Toll-like receptors expressed on both of these cell types. Activated mature immune cells such as dendritic cells initiate adaptive immunity by educating tumor-specific T cells and secreting pro-inflammatory cytokines (TNF-α, IL-6, IL-12, IL-1β, IFN-β, etc.) that induce the secretion of DAMP proteins from tumor cells. TLR axis signaling in tumor cells induces the transcription of genes related to cell proliferation, chemotherapeutic resistance, invasion, and metastasis in tissues (e.g., lung tissue). In addition, tumors usually escape the immune response by altering the tumor microenvironment with immunosuppressive populations (Treg cells, MDSCs, M2 macrophages, etc.) and secretion of anti-inflammatory cytokines (IL-10, TGF-β, etc.).

Damage-associated molecular patterns (DAMPs), also known as danger-associated molecular patterns, are endogenous molecules released from dying cells that activate the immune system by interacting with pattern recognition receptors (PRRs)^1^. DAMPs contribute to host defense through inflammation induced by immune cells, though they are also related to autoimmune disease when chronically induced^2^. After chemotherapy or radiotherapy, tumor-derived DAMPs interact with Toll-like receptors (TLRs) that activate immune cells and produce inflammatory cytokines^3^. These pro-inflammatory cytokines mediate innate and adaptive immunity through autocrine secretion by immune cells. Moreover, these inflammatory cytokines directly induce tumor-derived secretory proteins that also stimulate paracrine secretion of inflammatory cytokines by immune cells. However, chronic inflammation ultimately increases the population of immunosuppressive cells in the tumor microenvironment and elevates the expression of immune checkpoint molecules that allow tumors to evade immune responses. Recently, it has been reported that tumor-derived DAMP proteins directly increase endothelial-to-mesenchymal transition (EMT), which is responsible for tumor metastasis^1,4^. Furthermore, TLR-overexpressing tumors have been shown to result in a poor prognosis for cancer patients^5^. Indeed, tumor-derived DAMP proteins bind to TLRs expressed on tumors and activate downstream signaling that is associated with tumor migration, invasion, and metastasis^6,7^. Here, we suggest that DAMPs are known to be associated with immune cells, but they could be important mediators between immune cells and tumors because tumor-derived secretory proteins can induce the production of pro-inflammatory cytokines by immune cells and directly increase the aggressive characteristics of tumors through TLRs (Fig. 1).

## DAMP proteins released from dying cells

DAMPs are danger signals that are released from dying or necrotic cells and are also actively secreted from cells by external stimulation (e.g., high-mobility group box 1 (HMGB1) protein)^8–11^. Chemotherapy or radiotherapy usually induces the release of DAMP proteins following tumor apoptosis or necrosis^12–15^. Tumor-derived DAMP proteins are expressed in various tumor types (Table 1). As a well-known DAMP molecule, HMGB1 can be passively released from damaged cells or actively secreted from activated immune cells through a leaderless secretory pathway^16–19^. Tang et al.^19^ reported that HMGB1, present in almost all metazoans and plants and first identified as a chromatin-associated protein, is a highly conserved nuclear protein that acts as a chromatin-binding factor that binds DNA and facilitates the assembly of transcriptional proteins on specific DNA targets. Moreover, HMGB1 has been implicated in disease states, including sepsis, ischemia-reperfusion, arthritis, meningitis, neurodegeneration, aging, and cancer^20–26^. In addition, it is associated with all of the central hallmarks of cancer and is released from tumor cells when induced by chemotherapy or radiotherapy^27–29^. Napolitano et al.^30^ assessed the serum levels of HMGB1 to reliably distinguish malignant mesothelioma patients, asbestos-exposed individuals, and unexposed controls. Total HMGB1 was significantly increased in malignant mesothelioma patients and asbestos-exposed individuals compared with healthy controls. Naumnik et al.^31^ investigated the prognostic role and effects of chemotherapy on HMGB1 serum levels in patients with advanced-stage non-small cell lung cancer (NSCLC). After four cycles of chemotherapy, the mean serum HMGB1 levels in peripheral blood samples were significantly higher in patients with advanced NSCLC than in controls. Moreover, it has been shown that a DAMP protein, HMGB1, functions in a manner depending on the oxidative status. Tang et al.^32^ noted that HMGB1 is integral to oxidative stress and downstream apoptosis or survival. When HMGB1 accumulates at sites of oxidative DNA damage, it can lead to DNA repair. Both reduced and oxidized HMGB1 have been shown to perform different functions in extracellular signaling and regulation of the immune response through Toll-like receptors. HMGB1 released from injured or necrotic cells or secreted from inflammatory cells triggers immune responses after binding receptor for advanced glycation end products (RAGE) or Toll-like receptor (TLR)-2 or TLR4, triggering receptor expressed on myeloid cells-1 (TREM-1) and CD24, which stimulates cell migration, cell proliferation, and cell differentiation.

**Table 1:** The expression of tumor-derived DAMP proteins in various tumor types.

DAMPs	Tumor type	Function	Reference
HMGB1	Prostate cancer Malignant mesothelioma Hepatocellular carcinoma Colon carcinoma Pancreatic adenocarcinoma Mammary carcinoma Fibrosarcoma Lymphoma Osteosarcoma Melanoma Lewis lung carcinoma	Inducing adaptive immune response Cell migration and proliferation Angiogenesis Metastasis, chemoresistance Escape of apoptosis Tumor-antigen processing Presenting tumor antigen Trigger sterile inflammation Progression of tumors Neutrophil recruitment Increase autophagy	^119–131^
S100s	Breast cancer Fibrosarcoma Neuroblastoma Colon cancer Colorectal adenocarcinoma	Cell growth Myeloid cell infiltration Epidermal hyperplasia Secretion of pro-inflammatory cytokines Metastasis	^131–136^
HSPs	Breast cancer Colorectal cancer Melanoma	Involve in protein folding Activate immune response Metastasis	^39–41,46,137^
Histones	Pancreatic cancer Lung cancer	Chromatin remodeling Gene transcription Neutrophil migration Endothelial injury	^48,49,52,138^
PAUF	Pancreatic cancer Ovarian cancer	Pancreatic tumor development TLR4 stimulation Tumorigenesis Chemoresistance	^5,7,54,87^
API5	Adenocarcinoma Cervical cancer Breast cancer	Apoptosis inhibition Activating immune cells	^57–59^
RPS3	Adenocarcinoma Colorectal cancer Melanoma	Translation initiation Repair of UV-induced DNA damage Adjuvant for DC-based vaccine	^63,66,67^

Bresnick et al.^33^ reported that S100 protein family members, 21 proteins with a high degree of structural similarity, act as Ca^2+^ sensors that can translate fluctuations in intracellular Ca^2+^ levels into a cellular response and participate in a wide range of biological processes, such as proliferation, migration and/or invasion, inflammation, and differentiation. The function of S100 protein family members as DAMPs has been detected in the extracellular space, as well as in serum, urine, sputum, cerebrospinal fluid, and feces, and is generally associated with specific diseases, such as cancer^33–36^. The exact secretion mechanism of S100 proteins is still unclear, as they lack a sequence responsible for secretion through the classical endoplasmic reticulum (ER)-Golgi pathway^33^. Harpio et al.^34^ noted that circulating S100B plays a role in the decision to switch treatment regimens. The concentration of circulating S100B predicts the duration of survival in melanoma patients and are very sensitive for the detection of metastatic growth of malignant melanoma, particularly at stage IV of the disease. Pedrocchi et al.^37^ reported that the S100 protein level is elevated in the human breast cancer cell lines MDA-MD-231 and HS-578T. Cross et al.^38^ described how S100 protein family members, including S100A6, S100A8, S100A9, and S100A11, are expressed in breast cancer as well as other common cancers. In addition, S100A11 may have an effect on the proliferation of cancer cells through nucleocytoplasmic translocation.

Heat shock proteins (HSPs), a large family of molecular chaperones that are involved in protein folding, are overexpressed in various types of tumors and indicate a poor prognosis and increased resistance to therapies^39–42^. Released from dying tumor cells, they can also function as DAMP proteins to activate the immune system. In fact, the serum concentration of HSPs is elevated in cancer patients^43–45^. Fanelli et al.^39^ reported that the serum levels of the HSP Mr 27,000 (Hsp27), in breast cancer patients were higher than those in control patients. Chen et al.^46^ reported that serum concentrations of HSP90α in colorectal cancer patients were significantly higher than those in normal volunteers.

Histones are intranuclear cationic proteins that are present in all eukaryotic cells^47^. They act as DAMP proteins by activating the immune system through TLRs after being released into the extracellular space in response to injury or sepsis^14,48–51^. Chen et al.^48^ reported that histones are important in chromatin remodeling and gene transcription via posttranslational modifications. In addition, histones function as DAMPs when they translocate from the nucleus to the extracellular space, binding to receptors and activating various signaling pathways in a monomeric or multimeric manner. Moreover, ischemia/reperfusion (I/R) or drug-induced tissue injury results in sterile inflammation, and serum histone levels are significantly elevated in animal models of liver, kidney, lung, and brain injury. In addition, serum histone levels were found to be elevated in sepsis, and the H3 and H4 elements were the major components responsible for toxicity. In fact, the concentration of intracellular calcium is increased by the transient presence of exogenous histones in endothelial cells; histone administration in vivo causes neutrophil migration, endothelial injury and dysfunction, hemorrhage, and thrombosis, which finally results in animal death. Histones such as DAMPs can be released from dying or necrotic tumor cells^52^. Patwa et al.^49^ identified histone H4 in serum from pancreatic cancer patients.

Moreover, pancreatic adenocarcinoma upregulated factor (PAUF) is a ligand of TLRs that is involved in pancreatic tumor development^5,7,53^. PAUF is overexpressed in pancreatic cancer and acts as a DAMP protein, with resulting active secretion^54^. Kim et al.^54^ determined that PAUF is a novel upregulated secretory protein in pancreatic ductal adenocarcinoma—enhanced expression of PAUF mRNA and protein was observed in pancreatic cancer cell lines compared to normal cells. Furthermore, this group confirmed PAUF expression and secretion in lysates and culture medium by immunoreaction with anti-rhPAUF antibodies. Expression of PAUF was also confirmed in human pancreatic cancer tissues by immunohistochemical staining. As a ligand of TLR4, apoptosis inhibitor 5 (API5) is an apoptosis inhibitory protein that prevents apoptosis induced by the transcription factor E2F1 and interacts with a nuclear factor involved in apoptotic DNA fragmentation^55–57^. For instance, API5 is associated with diseases, including adenocarcinoma and cervical cancer^58,59^. It can be released from tumor cells and functions as a DAMP protein activating immune cells^57^. Cho et al.^58^ established that API5 is overexpressed in cervical cancer, with prognostic significance. By generating Kaplan–Meier plots, this group indicated that API5 expression is an important prognostic factor in human cervical cancer; high API5 expression reflected significantly shorter disease-free survival and overall survival times.

40S ribosomal protein S3 (RPS3) is a ribosomal protein component of the 40S subunit, which contains the domain responsible for translation initiation^60–62^. The RPS3 protein is involved in the repair of UV-induced DNA damage^63–65^. The expression levels of RPS3 in adenocarcinomas and adenomatous polyps were found to be higher than those in adjacent normal colonic mucosa^66^. Kim et al.^66^ reported that RPS3 is overexpressed in colorectal cancer cells, suggesting the relationship between this protein and tumorigenesis. This group showed that the secretion of dimeric RPS3 into the extracellular space and the secretion levels of RPS3 were obviously higher in malignant cancer cells than in normal cells. They demonstrated that RPS3 is secreted into cell culture medium via the ER-Golgi-dependent pathway, as detected by enzyme-linked immunosorbent assay (ELISA). Park et al.^67^ reported that RPS3 is a housekeeping protein that is expressed in all eukaryotic cells and is released from tumor cells by exposure to anticancer drugs. To this end, they specifically confirmed the release of RPS3 from B16F1 and B16F10 melanoma cancer cell lines into culture medium through western blotting.

In summary, various DAMP proteins are released from dying cells by tissue injury or chemo-/radiotherapy-induced apoptosis, and these DAMP proteins are associated with various diseases and functions through multiple signaling pathways.

## Interaction of tumor-derived DAMP proteins with TLRs

DAMPs have been described as ligands for TLRs, known as PRRs, which initiate an inflammatory response^68–72^. The biological structure of TLRs comprises an extracellular transmembrane domain composed of multiple leucine-rich repeats (LRRs) and an intracellular signaling domain featuring a Toll-IL-1 receptor (TIR) domain^73–75^. Regarding intracellular signaling, myeloid differentiation factor 88 (MyD88), MyD88 adapter-like (Mal) protein, TIR-related adapter protein inducing IFN-β (TRIF), and TRIF-related adapter molecule (TRAM) are involved in activating immune cell responses^76^. TLRs are expressed in immune cells, such as macrophages and DCs, which are involved in innate immunity^77–79^ (Table 2). Innate immune cells produce inflammatory cytokines, and costimulatory molecules are increasingly expressed after a ligand, such as a DAMP protein, activates TLR signaling^80,81^. In particular, TLR2 and TLR4 are receptors triggered by a variety of endogenous DAMPs to induce an inflammatory response^81^. As mentioned earlier, HMGB1 is actively secreted from inflammatory immune cells or passively released by necrotic cells to signal tissue damage and initiate immune responses by binding to receptor for advanced glycation end products (RAGE), TLR2, or TLR4. Gary et al.^18^ reported that HMGB1 is a DNA-binding nuclear protein that is released actively by cytokine stimulation and passively during cell death and has been implicated in several inflammatory diseases, as it is a prototypical DAMP protein. Furthermore, this group identified the relationship between HMGB1 and TLR4, in which HMGB1 plays an important role in the activation of DCs through TLR4 for efficient presentation of tumor antigens from dying cells. Moreover, stimulation of RAGE-transfected HT1080 cells with HMGB1 induces the activation of ERK1/2 signaling downstream of RAGE. Wang et al.^82^ described how HMGB1 is associated with TLR4 in drug-induced damage-associated lethal hepatitis. In addition, the serum HMGB1 concentration was found to rise after treatment with acetaminophen. The interaction between HMGB1 and TLR4 was confirmed by stimulating macrophages from TLR4^+/+^ or TLR4^−/−^ mice with soluble HMGB1; the production of IL-23 increased only with TLR4^+/+^ macrophages. Curtin et al.^83^ reported that HMGB1 mediates endogenous TLR2 activation in brain tumors. The supernatants from dying Ad-TK + GCV-treated GL26 tumor cells indicated that TLR2-dependent NFκB activation was inhibited when tumor cells were treated with glycyrrhizin, an antagonist of HMGB1.

**Table 2:** The expressions and functions of TLRs in various immune cells.

Immune cells	TLR type	Function	Reference
CD8^+^ T cell	TLR 2	Cytokine secretion Enhance proliferation	^139,140^
CD4^+^ T cell	TLR 2, 5, 7, 8	Cytokine secretion Enhance proliferation	^139,140^
γδ^+^ T cell	TLR 2, 3	IFNγ production	^139,140^
Treg	TLR 2/8 TLR 2/5	Reduced suppression Enhance suppression	^139,141^
mDC	TLR 1–10	Produce iNOS, TNF-α, IL-1β Upregulation of CD40, CD80, CD86, CCR7	^142–144^
pDC	TLR 7, 9, 10	Upregulation of CD40, CD80, CD86, CCR7 Produce IFNα	^143,144^
Macrophage	TLR 1–9	Produce pro-inflammatory cytokines	^142,145^
Mast cell	TLR 2, 4, 6, 8	Produce pro-inflammatory cytokines	^142,146,147^
Neutrophil	TLR 1–4, 6, 7, 9	Produce TNF-α ROS generation Increase survival	^142,148^
B cell	TLR 2–4, 6, 9	Secrete antibodies Development and differentiation	^142,149^

Alarmin eosinophil-derived neurotoxin (EDN) is known to use TLR2 as its receptor for activation of immune cells^84^. Yang et al.^84^ reported that EDN acts as an alarmin and activates TLR2-MyD88 signaling in dendritic cells. Furthermore, EDN was observed to induce an increase in luciferase activity in HEK293 cells expressing TLR2. In addition, the production of IL-6 in response to EDN was decreased in supernatants from TLR2^−/−^ DCs.

HSPs, as described previously, are released by necrotic cells or secreted via nonclassical pathways to convey a danger signal to surrounding cells, which is followed by binding to TLR2 or TLR4 to induce inflammation. Zhao et al.^85^ determined that heat-shock protein 60 (hsp60) induces IL-8 via the TLR2 and MAPK pathways in human monocytes. The concentration of NK-κB in NOMO1 cells was significantly reduced by pretreatment with an anti-TLR2 antibody before stimulation with recombinant hsp60 protein. Roelof et al.^86^ identified heat shock protein B8 (HSP22) as a novel TLR4 ligand and its involvement in the pathogenesis of rheumatoid arthritis. DC maturation by HSPB8 stimulation was inhibited in the presence of a TLR4 antagonist, and the expression levels of CD80, CD83, CD86, and MHC-II were decreased.

PAUF has been identified as an endogenous ligand of TLR2 and TLR4. Park et al.^7^ confirmed the specificity of the PAUF-TLR2 interaction, where PAUF induces ERK phosphorylation and activates the IKK-β-mediated TPL2/MEK/ERK signaling pathway through TLR2. Furthermore, TLR2 coimmunoprecipitated with PAUF formed by glycosylation, and preincubation of THP-1 cells with an anti-TLR2 antibody resulted in a significant decrease in the level of PAUF protein binding to TLR2. Kang et al.^87^ reported that PAUF functions as an adjuvant by activating DCs through TLR4. This group demonstrated PAUF-induced activation and maturation of DCs with activation of NF-κB through the TLR signaling pathway. To confirm the dependency of TLR4 and PAUF, the binding affinity of the PAUF protein for TLR4 was evaluated by a Blitz assay, and the levels of pro-inflammatory cytokines secreted from DCs lacking TLR4 were found to be reduced after treatment with PAUF.

Apoptosis inhibitor 5 (API5), which is overexpressed in many types of tumors, interacts with TLR4 and induces an inflammatory response. Kim et al.^57^ established that API5 activates antigen-presenting cells in a TLR4-dependent manner. Via western blotting, this group confirmed the release of API5 from chemically stressed cells of various murine and human cancer cell lines. Immunoprecipitation analysis revealed that API5 binds to TLR4 but not TLR2, and luciferase activity was increased in HEK293 cells after PAUF treatment. Moreover, activation and maturation of mouse and human DCs were observed after treatment with the PAUF protein, but the results were reversed in DCs lacking TLR4.

Furthermore, ribosomal family proteins have been shown to activate immune cells after binding to TLR4 and to induce inflammation within the tumor microenvironment. Park et al.^67^ reported that RPS3, a DAMP protein, is a novel adjuvant for DC-based vaccines, inducing maturation and activation of DCs through TLR4. The interaction between RPS3 and TLR4 was confirmed by a Blitz assay, and luciferase activity was found to be increased in HEK293 cells. Moreover, after treatment with RPS3, the maturation and activation of TLR4^−/−^ DCs were reduced compared to those of TLR4^+/+^ DCs.

Therefore, DAMP proteins are released from dying cells or tumor cells after chemotherapy or radiotherapy, and they bind and activate immune cells through TLRs to convey danger signals by initiating inflammatory immune responses.

## DAMP proteins act as mediators between immune cells and tumors through TLRs

DAMP proteins from dying or apoptotic cells can both activate immune cells to produce pro-inflammatory cytokines and stimulate tumor cells to be aggressive by promoting metastasis. These two cell subsets can be affected by TLRs reacting with DAMP proteins. Therefore, DAMP proteins can act as mediators between TLR-expressing immune cells and tumors.

Chemotherapy and radiotherapy induce tumor recurrence and metastasis even though most tumor cells are destroyed by apoptosis^13,88,89^. Moreover, tumor cells express functional TLRs that play important roles in their proliferation, apoptosis resistance, invasion, and metastasis^90–92^ (Table 3). Volk-Draper et al.^93^ reported that paclitaxel therapy promotes breast cancer metastasis via TLR4 signaling. The levels of inflammatory cytokines were increased in nab-PXL-treated TLR4^+/+^ tumors compared to untreated and nab-PXL-treated TLR4^−/−^ tumors. In fact, various types of cancer involve overexpression of TLRs, with poor prognosis in patients. Cammarota et al.^94^ observed that TLR4 expression is a potential prognostic marker in colorectal cancer, and the expression of TLR4 was associated with adenocarcinoma in human samples and a murine model. Increased TLR4 expression in the stromal compartment was associated with a significantly increased risk of disease progression, and significant tumor relapse occurred earlier in colon cancer patients with very high levels of TLR4 than in those with lower expression levels.

**Table 3:** The expressions and functions of TLRs in various tumors.

Tumor type	TLR type	Function	Reference
Ovarian cancer	TLR 2–5, 9	Immunosuppression Tumor growth Increased migration Resistance to chemotherapy	^6,150–152^
Cervical cancer	TLR 3–5, 7, 9	Tumorigenesis Tumor growth Resistance to chemotherapy	^152–155^
Lung cancer	TLR 2–4, 9	Prolong cancer cell survival Immune escape Apoptosis resistance Tumor metastasis	^104,156,157^
Colorectal cancer	TLR 2–5, 9	Tumorigenesis Antitumor activity Cancer proliferation Angiogenesis Inhibit tumor necrosis	^158–162^
Melanoma	TLR 2–4	Tumor migration Tumor progression Prolong cancer cell survival	^163,164^
Breast cancer	TLR 2–4, 9	Tumor invasion	^105,165^
Brain cancer	TLR 2, 4	Tumor progression Tumor metastasis	^166,167^
Prostate cancer	TLR 4, 9	Tumor invasion Carcinogenesis	^168,169^
Gastric cancer	TLR 2, 4, 5, 9	Tumor growth, invasion, and metastasis Angiogenesis Attenuate antitumor activity	^170–172^

TLRs are conventionally known to be involved in the host immune response; however, it has recently been shown that TLRs are also expressed in tumor cells and influence tumor progression^95–98^. Therefore, DAMP proteins released from tumor cells can directly induce tumors to be aggressive to promote migration, invasion, and metastasis through TLRs. Moreover, TLR signaling facilitates evasion of immune surveillance in the tumor microenvironment and contributes to resistance to anticancer drugs^99–101^. Kelly et al.^91^ and Dan et al.^102^ reported that activation of TLR4 signaling promotes the growth of ovarian cancer with resistance to chemotherapy and leads to significant increases in the levels of X-linked inhibitor of apoptosis and phosphorylated AKT. Jego et al.^103^ and He et al.^104^ described how tumor cell apoptosis is inhibited through TLR signaling in lymphoma and lung cancer. Merrell et al.^105^ reported that the invasion of the breast cancer cell line MDA-MB-231 was promoted by increased matrix metalloproteinase activity after activation by a TLR9 agonist. Thus, activation of TLRs expressed by tumor cells is associated with tumor aggressiveness. As components of the host immune system, chemo-/radiotherapy-induced DAMP proteins activate immune cells through TLRs to secrete pro-inflammatory cytokines, thereby clearing T-cell trafficking into the tumor microenvironment and ultimately clearing tumor cells. These pro-inflammatory cytokines induce autocrine secretion by immune cells in order to boost inflammation, which can result in autoimmune disease^106^. Moreover, these cytokines can induce paracrine secretion of secretory proteins by tumor cells, which can activate tumor cells through TLR signaling. Thus, DAMP proteins are associated with immune responses and tumor progression, and identification of DAMP proteins released from tumors is necessary to overcome autoimmune diseases or chronic inflammatory disorders such as atherosclerosis and arthritis^107^.

## Inflammation-induced immunosuppressive populations in the tumor microenvironment

Innate immunity and adaptive immunity are involved in host defense against tumor development^108^. First, innate immune cells, such as macrophages and DCs, recognize tumor antigens and deliver them to T cells to activate adaptive immune responses^109^. During the defense against tumors, immune cells produce pro-inflammatory cytokines such as TNF-α, IL-6, and IL-1β, and tumor-specific T cells produce perforin and granzyme B to clear tumor cells^110^. However, tumors usually escape the immune response by altering their microenvironment with immunosuppressive populations such as M2 macrophages, myeloid-derived suppressor cells (MDSCs), and regulatory T cells (Tregs)^111–113^. These immune cells produce suppressive cytokines (e.g., IL-10 and TGF-β) that render tumor cells aggressive and exhaust CD8^+^ T cells^114^. In fact, chemo-/radiotherapy-induced inflammation results in a suppressive tumor microenvironment that induces downregulation of host immune responses^115^. Moreover, DAMP proteins released from tumors can activate inhibitory immune cells that express TLRs. Song et al.^116^ reported that PAUF is a DAMP protein that is involved in pancreatic tumorigenesis and metastasis and enhances tumor-infiltrating MDSC functional activity via the TLR4-mediated signaling pathway. Moreover, Parker et al.^117^ noted that HMGB1 enhances immune suppression by facilitating the differentiation and suppressive activity of MDSCs. Liu et al.^118^ reported that knockdown of HMGB1 induces a decrease in regulatory T cells in tumors and restores CD8^+^ T-cell function. In conclusion, chronic inflammation induces a tumor microenvironment comprising immunosuppressive populations, where DAMP proteins activate TLR signaling expressed by inhibitory immune cells that are polarized toward suppressive characteristics.

## Concluding remarks

Tumor-derived DAMP proteins are functional in both immune cells and tumors. In immune cells, identification of DAMP proteins that bind to TLRs is important for inducing inflammatory responses. For example, DC-based vaccines require novel adjuvants that induce the maturation and activation of DCs to secrete inflammatory cytokines. In fact, DAMP proteins binding to TLR4 (e.g., HMGB1, S100, HSPs, API5, and RPS3) have been used as adjuvants for DC-based vaccines, affecting tumor prevention, treatment, and survival. In contrast, tumor progression and metastasis are associated with the inflammatory response based on the interaction between TLR signaling and DAMP proteins. Regulation of these DAMP proteins in tumors can reduce excessive inflammation to create an immunogenic tumor microenvironment. For example, neutralizing antibodies against specific DAMP proteins could be used for tumor treatment in combination with chemotherapy or radiotherapy to prevent immune escape. In conclusion, various DAMP proteins are released from tumor cells after apoptosis and are ligands of TLRs that initiate an immune response. As mediators between immune cells and tumors, DAMP proteins must be identified and applied for immunotherapy and cancer treatment.
